# *Caenorhabditis elegans* for rare disease modeling and drug discovery: strategies and strengths

**DOI:** 10.1242/dmm.049010

**Published:** 2021-08-09

**Authors:** Peter A. Kropp, Rosemary Bauer, Isabella Zafra, Carina Graham, Andy Golden

**Affiliations:** Laboratory of Biochemistry and Genetics, National Institute of Diabetes and Digestive and Kidney Diseases, National Institutes of Health, Bethesda, MD 20892, USA

**Keywords:** *C. elegans*, Disease modeling, Drug screens, Genetic screens, Patient-specific alleles

## Abstract

Although nearly 10% of Americans suffer from a rare disease, clinical progress in individual rare diseases is severely compromised by lack of attention and research resources compared to common diseases. It is thus imperative to investigate these diseases at their most basic level to build a foundation and provide the opportunity for understanding their mechanisms and phenotypes, as well as potential treatments. One strategy for effectively and efficiently studying rare diseases is using genetically tractable organisms to model the disease and learn about the essential cellular processes affected. Beyond investigating dysfunctional cellular processes, modeling rare diseases in simple organisms presents the opportunity to screen for pharmacological or genetic factors capable of ameliorating disease phenotypes. Among the small model organisms that excel in rare disease modeling is the nematode *Caenorhabditis elegans*. With a staggering breadth of research tools, *C. elegans* provides an ideal system in which to study human disease. Molecular and cellular processes can be easily elucidated, assayed and altered in ways that can be directly translated to humans. When paired with other model organisms and collaborative efforts with clinicians, the power of these *C. elegans* studies cannot be overstated. This Review highlights studies that have used *C. elegans* in diverse ways to understand rare diseases and aid in the development of treatments. With continuing and advancing technologies, the capabilities of this small round worm will continue to yield meaningful and clinically relevant information for human health.

## Abstract (precis)

**Summary:** Rare disease research has benefited from the generation of accurate genetic models. Here, the breadth of capabilities in rare disease modeling with *C. elegans* is emphasized through discussion of individual research stories.

## Introduction

The microscopic nematode *Caenorhabditis elegans* is a popular model organism thanks, in no small part, to the excellent molecular genetics and cell biology tools available for understanding processes relevant to humans. *C. elegans* was the first multicellular organism to have a fully sequenced genome (The C. elegans Sequencing Consortium, 1998), making it one of the premier genetic models available to researchers. Further, *C. elegans* was foundational in the discovery and development of RNA interference (RNAi) (Fire et al., 1998; Sugimoto, 2005), one of the most useful tools for assessing gene function and performing genetic screens. Most recently, the advent of CRISPR/Cas9 gene editing has allowed the facile generation of precision-modified alleles in *C. elegans* (Paix et al., 2015, 2016, 2017a,b), which are particularly beneficial in disease modeling. Researchers can precisely insert or delete specific sequences, modify single bases and even replace *C. elegans* genes with their human orthologs. These strategies have accelerated and improved the ability of researchers to investigate disease etiology and treatment. This Review will highlight some of the experimental approaches and strategies used to model rare human diseases in *C. elegans*.

Undeniably, the most essential aspect of modeling genetic diseases is the ability to accurately study the gene, and gene product, of interest. *C. elegans* and humans share greater than 50% of their genes (Kim et al., 2018), and, of the genes implicated in human disease, this percentage rises even higher as can be achieved by cross-referencing with disease allele databases such as the Online Mendelian Inheritance in Man (OMIM; https://www.ncbi.nlm.nih.gov/omim). Beyond conservation of individual genes, one benefit of studying a particular gene in *C. elegans* is the comparatively low number of paralogs, which are genes within the same organism that have complementary function. A gene family in humans often has numerous paralogs, such as the multiple paralogs of *RAS* in the human genome (Bos, 1988). The *C. elegans* gene *let-60* is the only *KRAS* ortholog, thus simplifying the study of the whole *RAS* family (Beitel et al., 1990; Han and Sternberg, 1990). This genetic simplicity thereby allows for more focused studies into gene and protein function, a vital aspect of disease modeling. Another key component of disease modeling is the ability to accurately assess meaningful and tractable phenotypes. Although humans and *C. elegans* are quite physically divergent, many cellular processes are well conserved, thereby allowing for direct functional studies (Barr, 2005). Further, molecular pathways, particularly cell signaling, are extremely well conserved from *C. elegans* to humans. Phenotypes do not have to precisely match between nematodes and humans for important mechanistic discoveries to be made with *C. elegans* (Golden, 2017; McGary et al., 2010). This point is illustrated by cases such as polycystic kidney disease investigated in the male *C. elegans* tail (O'Hagan et al., 2014), the RTK-Ras-MAPK pathway modeled in the hermaphrodite vulva (Kornfeld et al., 1995; Sundaram, 2013; Sundaram and Han, 1995), and many neurodegenerative disease phenotypes investigated with motility and bending assays that are quite unlike human motion (Caldwell et al., 2020). Lastly, *C. elegans* provides an excellent model in which to perform screens, genetic or pharmacological, that are unmatched in other model organisms. Although both *Drosophila* and zebrafish provide apt systems for high-content screens, they do not match the ability of *C. elegans* for high-throughput analysis (Patten et al., 2016). This fact is further illustrated by the advent of multiple protocols to perform such assays, including the more recent use of microfluidic platforms to improve high-throughput screening (Atakan et al., 2020; Kinser and Pincus, 2017; Lucanic et al., 2018; O'Rourke et al., 2009).

The utility of *C. elegans* for rare disease modeling is underscored by their inclusion in model organism screening centers (MOSCs), a component of the newly expanded Undiagnosed Disease Network (UDN; https://undiagnosed.hms.harvard.edu/). Originally started within the National Institutes of Health, the Undiagnosed Disease Program (UDP) was established to diagnose diseases, presumably genetic in nature, in individuals with novel symptomology and who have endured the diagnostic odyssey of searching for answers from clinicians without success (Gahl et al., 2016). The UDP was expanded to 12 sites in the United States, thus giving rise to the UDN.

The genetic diagnosis process within the UDN includes whole-genome sequencing, which frequently identifies variants in multiple genes. These are then deployed for candidate testing to assess genetic causation of the disease. Vetting candidate variants via examination in a model organism allows for the linkage of the causative gene and variant to the disease phenotype. Because this method is so powerful, ∼20% of candidate variants are currently evaluated in model systems including zebrafish, *Drosophila* and *C. elegans* at MOSCs (Gahl et al., 2016). The results from these studies will hopefully continue to improve the outcomes of individuals who seek the help of the UDN. Such approaches are not limited to the UDN and associated MOSCs. Rare disease-focused model organism networks exist outside of the United States and include the UDN International (UDNi; https://www.udninternational.org/), the Canadian Rare Diseases Models and Mechanisms Network (http://www.rare-diseases-catalyst-network.ca/) and the European Solve RD (http://solve-rd.eu/). Collectively, these networks will hopefully bring light and understanding to many rare diseases.

This Review will present the work of individual groups as vignettes to illustrate the potential of *C. elegans* in rare disease modeling. Each vignette will cover a different strategy for disease modeling and, where possible, emphasize the impact that these studies have had on patients. We will highlight studies that have worked with the *C. elegans* ortholog of a human disease gene, as well as those that have opted to express the human gene in the animal. We also include a study that investigated a human gene for which no *C. elegans* ortholog exists in order to identify potential novel drug targets. Two of the vignettes in this Review successfully identified drugs capable of suppressing the disease phenotypes in *C. elegans* and other model organisms. One of these drugs is currently in clinical trial for treatment of the human disease.

## Genetic modulation to model diseases in *C. elegans*

### Transgenic expression of a disease-associated variant

The first vignette illustrates the power of transgenesis to model diseases in *C. elegans*. Multiple rare conditions have been associated with pathogenic variants of *NALCN*, which produces the nonspecific ion leak channel NALCN*.* Although low expression of NALCN can be detected in non-neuronal tissues, it is predominantly expressed in the brain, where it is responsible for Na^+^ leak conductance important for the electrical excitability of neurons (Al-Sayed et al., 2013; Köroğlu et al., 2013; Lu et al., 2007). Consequently, individuals with *NALCN* mutations develop neurodegenerative symptoms. Multiple studies have reported children with hypotonia, facial dysmorphia and global developmental delay related to recessive loss-of-function mutations in *NALCN*, in which the channel fails to open (Al-Sayed et al., 2013; Köroğlu et al., 2013). A successful collaboration between clinicians and *C. elegans* researchers characterized a novel gain-of-function mutation in *NALCN* (Aoyagi et al., 2015). This study, led by Jacques Michaud, Sainte-Justine Research Center (Montreal, QC, Canada), and Mei Zhen, Lunenfeld-Tanenbaum Research Institute (Toronto, ON, Canada), reported a child with intellectual disability, ataxia, congenital arthrogryposis and a heterozygous arginine-to-glutamic acid substitution at residue 1181 [R1181Q] in NALCN (Aoyagi et al., 2015). That this substitution was heterozygous indicated that it was genetically dominant and suggested that it resulted in a gain of function for the NALCN channel. Typically, gain-of-function variants of ion channels increase permeability, and thus ion conductance, because the channel stays open even when it should not. Such persistent opening is deleterious to the function of the cell or tissue in which the channel is expressed. Therefore, it was not surprising that this individual with a gain-of-function mutation had a clinical presentation that was different from the previously identified loss-of-function *NALCN* mutations. In the effort to understand the consequences of the NALCN [R1181Q] variant, researchers pursued studies in *C. elegans* to determine whether such a dominant missense variant in NALCN could be pathogenic.

**Fig. 1.**
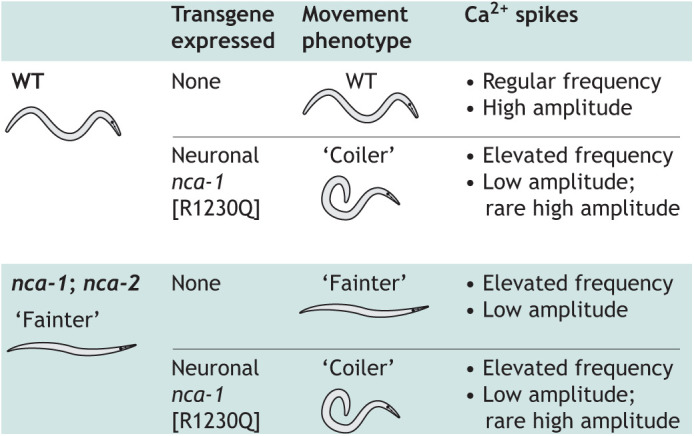
**Determination of the dominant effects of the *nca-1* [R1230Q] variant in sodium leak channel-related neurodegeneration.** Wild-type (WT) *C. elegans* have stereotypical sinusoidal movement, which, when perturbed, indicates neuromuscular defects. *nca-1; nca-2* double-null animals present with the ‘fainter’ phenotype due to reduced synaptic transmission at neuromuscular junctions. Animals with pan-neuronal expression of a transgene expressing the dominant *nca-1* [R1230Q] variant present with the ‘coiler’ phenotype characteristic of elevated synaptic transmission at neuromuscular junctions regardless of genetic background. Transgene expression also affected Ca^2+^ spikes measured from the AVA interneuron. All data from Aoyagi et al. (2015).

There are two functionally redundant *NALCN* orthologs in *C. elegans*: *nca-1* (also known as *unc-77*) and *nca-2*. Both *nca-1* and *nca-2* are expressed in motor neurons, where, like *NALCN*, they contribute to the Na^+^ leak that is important for electrical excitability and secretion of neurotransmitters (Yemini et al., 2013). Concurrent loss of *nca-1* and *nca-2* function results in a ‘fainter’ phenotype where animals fail to sustain sinusoidal movement due to decreased muscle contractility. Conversely, gain-of-function alleles of either gene alone result in a ‘coiler’ phenotype exemplified by exaggerated body bends due to increased muscle contractility (Gao et al., 2015; Xie et al., 2013; Yeh et al., 2008) (Fig. 1). The R1181 residue of human NALCN is conserved in both *C. elegans* NCA-1 and NCA-2, allowing for investigation of this specific patient variant. Employing a transgenic approach, Michaud, Zhen and colleagues used a pan-neuronal promoter to express NCA-1 carrying the orthologous substitution [R1230Q] in both wild-type (WT) and in *nca-1*; *nca-2* double-null animals. They determined that neuronal expression of the *nca-1* [R1230Q] substitution was sufficient to convert both WT and *nca-1*; *nca-2* double-null animals to the coiler phenotype (Aoyagi et al., 2015). Their results indicate that *nca-1* [R1230Q], and consequently its *NALCN* [R1181Q] ortholog, are genetically dominant and result in a gain of function for NCA-1 and NALCN channel activity.

Michaud, Zhen and colleagues further characterized the effect of the *nca-1* [R1230Q] on neuronal function by investigating Ca^2+^ spikes in the AVA interneuron. In WT animals, backward motion initiates Ca^2+^ spikes from this neuron. These spikes had reduced amplitude despite their elevated frequency in the *nca-1*; *nca-2* double-null animals (Fig. 1). However, Ca^2+^ spikes were highly irregular when the *nca-1* [R1230Q] transgene was expressed pan-neuronally (Aoyagi et al., 2015). Spikes in the transgenic animals were exaggerated in frequency, with many low-amplitude ones interrupted by rare and inconsistent high-amplitude spikes (Aoyagi et al., 2015). These results confirmed the electrophysiological impact of the *nca-1* [R1230Q] variant as a gain-of-function allele and exemplify how clinicians and *C. elegans* researchers collaborated to rapidly model a novel genetic variant. In the conclusions of their publication, the researchers speculated about verapamil, a US Food and Drug Administration (FDA)-approved Ca^2+^-channel blocker, as a potential therapy for treatment of gain-of-function variants in *NALCN* (Aoyagi et al., 2015). Future studies will have to determine the efficacy of verapamil in this context. This example illustrates the benefit of *C. elegans* for functional validation of a monogenic disease variant and led to the exploration of potential therapies. With the improving ease of transgenesis and gene editing, approaches such as the one described here could become increasingly common for modeling rare disease-causing variants.

### Modulating gene expression to evaluate disease modifiers

Although a rare disease may be monogenic, its severity can be modified by other factors, including the activity of other genes. Thanks to their facile genetic capabilities, *C. elegans* are a useful tool for evaluating the impact of genetic modifying factors that contribute to disease presentation. One such case is the motor neuron disease spinal muscular atrophy (SMA). SMA is caused by reduced expression of survival of motor neuron (SMN) protein, which is required for small RNA processing and, as is increasingly appreciated, endocytosis (Dimitriadi et al., 2010). Although there are two *SMN* paralogs in humans, *SMN1* and *SMN2*, only ∼10% of *SMN2* mRNAs are correctly spliced and produce functional protein due to a single silent mutation affecting an exonic splicing enhancer (Lefebvre et al., 1998; Lorson and Androphy, 1998; Monani et al., 1999). Most SMA-affected individuals carry a homozygous deletion of *SMN1* (Lefebvre et al., 1998), so *SMN2* is their only source of SMN protein and activity. As such, *SMN2* copy number, which naturally varies, is a primary determinant of SMA severity. However, some individuals carrying a homozygous *SMN1* deletion as well as low *SMN2* copy number are asymptomatic in spite of the fact that, genetically, SMA should be inevitable.

**Fig. 2.**
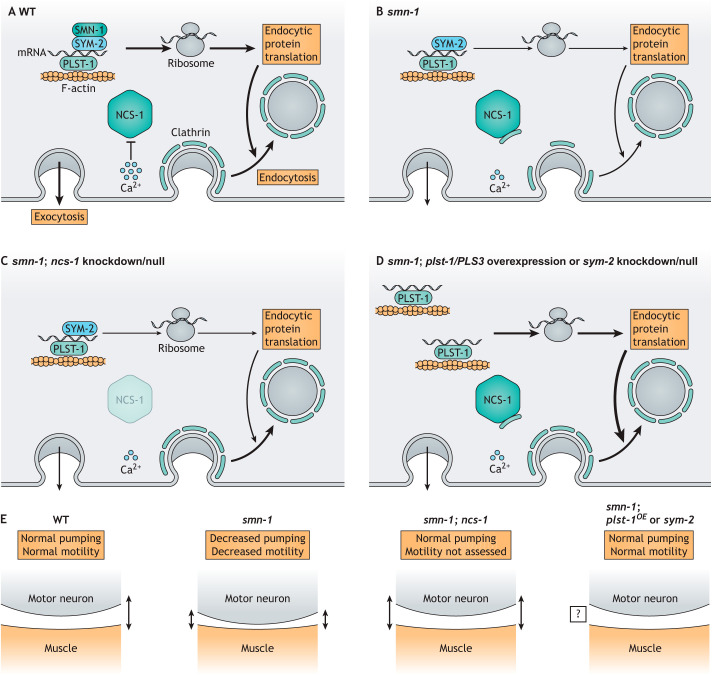
**Spinal muscular atrophy phenotypes suppressed by *C. elegans* modifiers.** (A) In WT animals, SMN-1, SYM-2 and PLST-1 exist in large protein complexes that also include F-actin and mRNAs encoding endocytic proteins. Translation of these mRNAs produces the proteins necessary for endocytosis. Independently, exocytosis results in localized elevations in Ca^2+^, which inhibits the activity of NCS-1. (B) In *smn-1* mutants, mRNA transport and translation are reduced. Exocytosis is also reduced, which decreases localized Ca^2+^ and allows NCS-1 to sequester clathrin, thereby further reducing the number of endocytic vesicles. These effects compound to reduce endocytosis. (C) Knockdown or deletion of *ncs-1* prevents clathrin sequestration, restoring the pool of clathrin available for endocytosis (Dimitriadi et al., 2010). (D) High-copy expression of the *C. elegans plst-1* or the human *PLS3* gene improves mRNA transport and translation, increasing the abundance of endocytic proteins (Walsh et al., 2020). (E) The *smn-1* mutants display impaired neuromuscular junctions (NMJs), as determined by reduced pharyngeal pumping and decreased motility. Data from zebrafish suggest that the impairment is due to reduced size of the synaptic cleft. Knockdown of *ncs-1* in the *smn-1* mutant background rescues the pharyngeal pumping phenotype presumably by restoring the appropriate synaptic cleft (Riessland et al., 2017). Motility was not assessed in the experiment. It is unclear how overexpression (OE) of *plst-1* or knockdown of *sym-2* affects the synaptic cleft, but either condition was sufficient to rescue pharyngeal pumping and motility in the *smn-1* background (Dimitriadi et al., 2010; Walsh et al., 2020).

Work led by Brunhilde Wirth, University of Cologne (Köln, Germany), has focused on identifying protective genetic background modifiers that could potentially explain the lack of symptoms in these individuals. Through a combination of experiments in human tissues and cell lines, as well as in mouse and zebrafish models of SMA, Wirth's group has identified both plastin 3 (PLS3) and neurocalcin delta (NCALD) as protective modifiers in SMA-affected individuals (Ackermann et al., 2013; Heesen et al., 2016; Oprea et al., 2008; Riessland et al., 2017). These two proteins are positive and negative regulators of neuronal endocytosis, respectively (Fig. 2A-D). Wirth and colleagues showed increased and decreased expression of PLS3 and NCALD, respectively, in subsets of SMA-affected individuals (Oprea et al., 2008; Riessland et al., 2017). These results suggested that enhanced neuronal endocytosis contributes to the milder SMA presentation in some patients, but further characterization was necessary.

Wirth and colleagues included a *C. elegans* model of SMA in their studies of NCALD and its ability to suppress SMA phenotypes. *C. elegans* has a single *SMN* ortholog, *smn-1*, leading to an SMA model in which *smn-1* loss-of-function mutants exhibit neurodegenerative phenotypes, including endocytic defects and reduced pharyngeal pump frequency (Dimitriadi et al., 2010). As *C. elegans* pharyngeal pumping is highly sensitive to neuromuscular disruptions, this phenotype is an easily assayed metric of neuronal dysfunction. To validate *NCALD* as a protective modifier, its *C. elegans* orthologous gene, *ncs-1*, was investigated through both RNAi-mediated depletion and a loss-of-function mutation. In both cases, reduced NCS-1 activity was sufficient to restore the pharyngeal pump frequency of *smn-1* mutants to normal levels, presumably through improved maturation of the synaptic cleft (Fig. 2E) (Riessland et al., 2017). These results, paired with findings from zebrafish (Oprea et al., 2008), mice (Oprea et al., 2008) and NSC34 mouse neuron-like cells (Riessland et al., 2017), confirmed the well-conserved relationship between SMN and NCALD or their orthologs in other species. Thus, Wirth and colleagues demonstrated the utility of *C. elegans* for physiological evaluation of genetic relationships using well-established tools such as RNAi or publicly available mutant strains.

Further work related to the SMA-suppressing factor PLS3 was performed by Anne Hart's group at Brown University (Providence, RI, USA). They characterized the *C. elegans* ortholog of PLS3, PLST-1, and confirmed its role in suppressing neurodegenerative phenotypes in the *smn-1* mutant SMA model. High-copy transgenic expression of either *plst-1* or human *PLS3* was sufficient to suppress the locomotion defects of the *smn-1* mutant (Fig. 2E) (Dimitriadi et al., 2010; Walsh et al., 2020). Thus, by using the ability to alter expression of the suppressing factor in *C. elegans*, Hart and colleagues could validate the neuromuscular effects of increased *PLS3*/*plst-1* expression.

Hart and colleagues continued their analysis of SMA suppressors by searching for novel factors that could modify the *smn-*1 neurodegenerative phenotypes. Building on the work of Wirth's group, who showed that SMN and PLS3 colocalize in large actin-containing bundles (Oprea et al., 2008), Hart and colleagues set up a genetic screen to identify additional genes that, when depleted, could suppress the neuromuscular defects caused by decreased SMN-1 levels (Walsh et al., 2020). They leveraged previously generated biochemical data of likely SMN-1 or PLST-1 interactors from *Drosophila* proteomics (Sen et al., 2013) to identify 11 candidate suppressors with existing loss-of-function alleles in *C. elegans*. All 11 candidates were analyzed in functional assays, but only loss of SYM-2, the ortholog of the human RNA-binding heterogeneous nuclear ribonucleoprotein F/H, suppressed the endocytic defects of *smn-1* mutant animals (Walsh et al., 2020). Further, the *sym-*2 loss-of-function allele ameliorated the locomotion defects of the *smn-1* mutant (Fig. 2E) (Walsh et al., 2020).

Finally, Hart's group evaluated the roles of *PLS3* and *sym-2* in endocytosis. The endocytosis of secreted proteins from the *C. elegans* pseudocoelom, typically scored using a fluorescent reporter, is a well-established method of assessing the function of endocytosis regulators. As the *smn-1* mutant fails to endocytose proteins from the pseudocoelom, this is a good strain for studying modifiers of that function. Transgenic overexpression of *PLS3* or RNAi knockdown of *sym-2* were both sufficient to improve endocytosis in the *smn-1* mutant (Fig. 2D) (Walsh et al., 2020). Thus, modulating the expression of *PLS3*/*plst-1* or *sym-2* led to suppression of several phenotypes observed in the *C. elegans* SMA model. Notably, the mechanism of phenotype suppression by *plst-1* and *sym-2* is markedly different from that by *ncs-1*. NCS-1 directly impairs endocytosis by sequestering clathrin, the protein necessary for membrane bending (Fig. 2B,C). PLST-1 and SYM-2 appear to both function in the mRNA processing and transport complexes in which SMN-1 is also present. Therefore, PLST-1 overexpression or SYM-2 knockdown can increase the processing and transport of mRNAs encoding endocytic proteins, thereby indirectly promoting endocytosis (Fig. 2D).

These studies demonstrate the utility of *C. elegans* for the identification and examination of genetic modifiers found in asymptomatic individuals with *SMN1* variants. Through the modulation of gene expression via transgenesis and/or RNAi, this work allows us to understand the mechanism and potential therapeutic targets of SMA in a simple and well-established system. Importantly, it also provides a blueprint for investigating genetic modifiers of other monogenic diseases.

### Determining the function of disease-associated genes

The next vignette illustrates the power of direct genetic engineering using CRISPR/Cas9 paired with transgenesis. NGLY1 deficiency is an ultra-rare autosomal recessive disease first characterized in 2012 and, as of writing this Review, 63 patients have been reported in the literature (Need et al., 2012; https://rarediseases.org/rare-diseases/ngly1-deficiency/). Symptoms and symptom severity vary, but nearly all comprehensively examined patients exhibit some form of movement disorder along with hypotonia and developmental delays (Cahan and Frick, 2019; Enns et al., 2014; Lam et al., 2017; Need et al., 2012; Pinto et al., 2020). A genetic diagnosis was made when the first patient underwent diagnostic whole-exome sequencing, revealing compound heterozygous mutations in the *NGLY1* gene, which encodes the protein N-glycanase (Need et al., 2012; Suzuki et al., 2016).

Prior to its implication in a novel disorder, N-glycanase was thought to play a relatively minor role in the endoplasmic reticulum (ER)-associated degradation (ERAD) pathway (Suzuki et al., 2016). N-glycan, a multi-sugar moiety, is added to the asparagine of an asparagine-X-serine/threonine motif (Asn-X-Ser/Thr) during protein folding (Kornfeld and Kornfeld, 1985). The N-glycan serves as a molecular record of failed protein folding because each time folding fails, a sugar moiety is enzymatically removed until the remaining glycan's structure becomes the substrate for the ER retrotranslocon and the protein is ubiquitinated for degradation (Vembar and Brodsky, 2008). N-glycanase is responsible for removing the N-glycan remains from the protein prior to proteasomal degradation (Suzuki et al., 2016; Vembar and Brodsky, 2008). When N-glycanase is nonfunctional, misfolded proteins that are not degraded accumulate in the cytoplasm, potentially forming aggregates that impede cellular function (Suzuki et al., 2016; Vembar and Brodsky, 2008). Over 12 NGLY1 deficiency-causing variants are known to reside within the highly conserved transglutaminase and mannose-binding domains; therefore, study of the *C. elegans* ortholog *png-1* represents a good potential model for this disease (https://rarediseases.org/rare-diseases/ngly1-deficiency/).

**Fig. 3.**
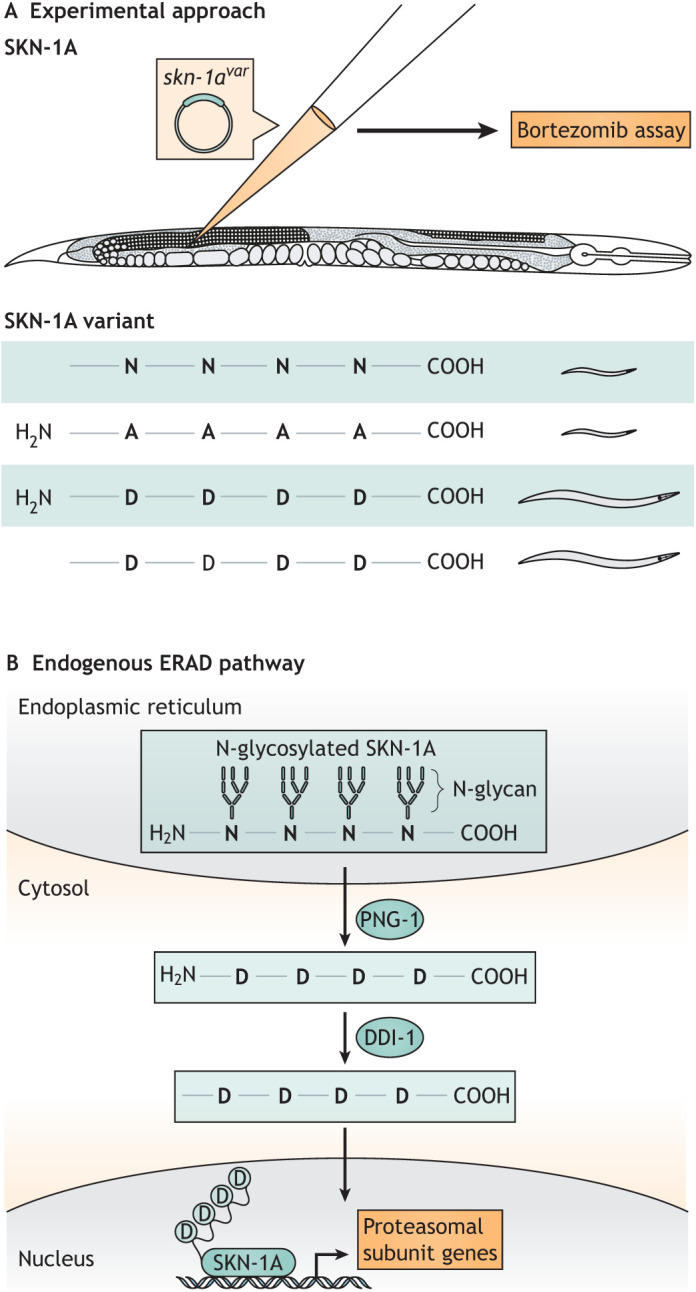
**Discovery of SKN-1A modification by PNG-1 and DDI-1 in the ultra-rare disease NGLY1 deficiency.** (A) To elucidate SKN-1A protein sequence editing and cleavage by PNG-1 and DDI-1, respectively, researchers injected variant *skn-1a* transgenes into *skn-1a* null *C. elegans* germlines. The resulting progeny were assayed for sensitivity to proteasomal stress via exposure to bortezomib (top). If sensitive to bortezomib, animals arrest as larvae. If bortezomib sensitivity was rescued by the *skn-1a* transgene, animals developed to adulthood (bottom). This approach allowed for the discovery of the endogenous modifications of SKN-1A in the ERAD pathway by PNG-1 and DDI-1, shown in B. (B) Deglycosylation by PNG-1 removes N-glycan groups and edits asparagine (N) to aspartate (D) residues. N-terminal cleavage by DDI-1 is also necessary for full activity of SKN-1A to activate transcription of genes encoding proteasomal subunits as part of the endoplasmic reticulum-associated degradation (ERAD) response. In NGLY1 deficiency, PNG-1/NGLY1 does not edit the SKN-1A/NRF1 asparagines to aspartates, so SKN-1A is incapable of activating the expression of its proteasomal subunit target genes. Impaired activation of proteasomal genes subsequently impairs ERAD, contributing to the neurodegeneration observed in NGLY1 deficiency. All data from Lehrbach and Ruvkun (2016) and Lehrbach et al. (2019).

We are highlighting this disease because the discovery of its mechanism originated as a purely basic research study to understand the regulation of the transcription factor SKN-1 and resulted in a rather dramatic revelation about the function of PNG-1/NGLY1 in *C. elegans* and humans. In 2016, Nicolas Lehrbach and Gary Ruvkun (Harvard Medical School, Massachusetts General Hospital, Boston, MA, USA) began to study the role of *C. elegans* PNGase in the ERAD pathway, particularly its relationships with the transcription factor SKN-1A and the protease DDI-1 (Lehrbach and Ruvkun, 2016). SKN-1A, an ER-associated isoform of SKN-1, is the *C. elegans* ortholog of mammalian Nrf1, which is constitutively expressed and subsequently degraded by the ERAD pathway (Lehrbach and Ruvkun, 2016; Radhakrishnan et al., 2014). However, in the event of proteasomal dysfunction, SKN-1A is not degraded and localizes to the nucleus, where it activates expression of a broad range of target genes, including those encoding proteasomal subunits (Lehrbach and Ruvkun, 2016). In this way, undegraded SKN-1A serves as both a sensor for proteasomal dysfunction and a participant in its remedy. However, SKN-1A activity also depends on the activity of PNG-1 and DDI-1 (Fig. 3) (Lehrbach and Ruvkun, 2016).

Lehrbach and Ruvkun first began to elucidate the relationship between SKN-1A, PNG-1 and DDI-1 after a screen for genetic suppressors of *skn-1*-dependent proteasome activation. Most of the suppressing mutations were within genes involved in the ERAD pathway, with *png-1* and *ddi-1* being over-represented in the screen (Lehrbach and Ruvkun, 2016). DDI-1 is a conserved aspartic protease implicated in the regulation of proteasomal function (Sirkis et al., 2006). *C. elegans* carrying either homozygous *png-1* or *ddi-1* null alleles displayed no obvious phenotypes. However, if treated with very low doses of bortezomib, a proteasome inhibitor commonly used to treat lymphoma, either null homozygote displayed larval arrest or lethality (Fig. 3A), indicating that *png-1* and *ddi-1* are necessary for overcoming proteasomal stress (Lehrbach and Ruvkun, 2016).

The authors used CRISPR/Cas9 to delete the SKN-1A DNA-binding domain and confirm that SKN-1A is responsible for regulating proteasome activity. Like *ddi-1* and *png-1* mutants, these *skn-1a* mutants failed to activate a reporter of proteasome activity, and the *skn-1a* and *ddi-1* double knockout did not enhance this phenotype, suggesting that both proteins work in the same pathway for conferring resistance to proteasomal stress (Lehrbach and Ruvkun, 2016). Despite confirming that SKN-1A is a target for DDI-1 cleavage in the context of proteasomal stress, this study determined that this cleavage was not sufficient to activate SKN-1A (Lehrbach and Ruvkun, 2016). Indeed, expressing a transgene that produced a truncated SKN-1A mimicking a DDI-1 cleaved SKN-1A in *skn-1* null animals was unable to rescue their sensitivity to bortezomib (Lehrbach et al., 2019). Given these results, the researchers turned their attention to the role of PNG-1 in SKN-1A activation using a *skn-1a* transgene, the protein product of which could not be glycosylated (Fig. 3). When expressed in *skn-1* null animals, this transgene was unable to rescue the bortezomib sensitivity seen in both the *skn-1* and the *png-1* null mutants (Lehrbach et al., 2019). This result suggested that retention of N-glycans is not the main factor in *png-1* mutants' sensitivity to proteasomal stress, and/or that glycosylation and subsequent deglycosylation is required for SKN-1A-mediated upregulation of proteasomal subunits' gene expression.

To ascertain why glycosylation is required for proteasomal stress resistance, a secondary function of PNG-1 was explored. When PNG-1 deglycosylates an asparagine residue, it also deaminates it into an aspartic acid. To evaluate the importance of this protein sequence editing, Lehrbach and Ruvkun engineered another SKN-1A transgene that replaced the four potential asparagine glycosylation sites with aspartates. Expression of this transgene in *skn-1a* null animals rescued the larval arrest upon exposure to bortezomib and extended the survival of adult animals on bortezomib-infused agar plates, suggesting that protein sequence editing rather than deglycosylation is the crucial role of PNG-1 in SKN-1A activation (Lehrbach et al., 2019). Expressing this modified transgene in a *png-1* null mutant background had similar results, suggesting that the artificial sequence editing of SKN-1A successfully compensated for complete loss of PNG-1 activity (Lehrbach et al., 2019). A third SKN-1A-producing transgene was developed that not only included the aspartate substitutions described above, but also mimicked cleavage by DDI-1. This transgene was sufficient to rescue all *skn-1*, *ddi-1* and *png-1* null phenotypes, suggesting that both DDI-1 cleavage and PNG-1-mediated protein sequence editing are required for SKN-1A transcription factor activity in response to proteasomal stress (Fig. 3B) (Lehrbach et al., 2019).

Based upon these findings, PNG-1/NGLY1 plays a critical role in the cell's ability to respond to proteasomal stress via production of new proteasomes, because the activity of SKN-1A depends upon protein sequence editing by PNG-1. These findings help to explain the accumulation of protein aggregates seen in *png-1* mutants. Although future work will have to confirm the sequence-editing role of the N-glycanase in mammalian systems, this work provides a direct link between editing SKN-1A, or its human ortholog NRF1, and the dysfunctional ERAD pathway observed in NGLY1 deficiency. Therefore, the use of *C. elegans* as a model organism has allowed researchers to shed light on a medical mystery, bringing a former housekeeping gene into the clinical spotlight and elucidating its role in protein sequence editing in an ultra-rare disease.

NGLY1 deficiency and PNG-1 have garnered the attention of other groups, who have used *C. elegans* and *Drosophila* to identify chemical suppressors of PNG-1 dysfunction (Iyer et al., 2019). As drug discovery with other *C. elegans* disease models is the focus of the next section, this investigation will not be covered in detail here. Yet, Iyer and colleagues successfully identified catecholamines and nonsteroidal anti-inflammatory drugs as potential classes of therapeutics for NGLY1 deficiency, thereby illustrating the benefit and utility of *C. elegans* for drug discovery.

## *C. elegans* for use in drug discovery

One of the many strengths of *C. elegans* as a model for human disease is its utility in screening pharmacological compounds for clinical use. *C. elegans* has the multicellular systems necessary for assessing the complex mechanisms of pathologies while simultaneously allowing for identification of off-target effects. The ability to grow large, synchronous populations in liquid culture represents a cost advantage over mammalian cell culture or organoids. Although large populations of zebrafish and flies can also be used for drug screening, obtaining synchronous populations of these organisms at the scale of *C. elegans* is more challenging and thus limits throughput. For these and many more reasons, *C. elegans* is an excellent system for drug screening and discovery for human diseases. Multiple reviews have covered its utility for diverse screening modalities (Ben-Yakar, 2019; Kim et al., 2017a; Sepúlveda-Crespo et al., 2020), and the vignettes presented herein are by no means an exhaustive overview of *C. elegans* capabilities.

### Identification of therapeutics for amyotrophic lateral sclerosis (ALS)

We highlight one example of the successful identification of multiple compounds in *C. elegans* to treat human disease. Alex Parker, Université de Montréal (Montreal, QC, Canada), has been using *C. elegans* to study the neurodegeneration associated with ALS. *C. elegans* is a particularly beneficial model for neurodegenerative diseases given its fully mapped neural connectome (Cook et al., 2019), ease of imaging individual neurons and well-characterized neural phenotypes (Hobert, 2003; Schafer, 2005; Yemini et al., 2013). Behavioral phenotypes, such as susceptibility to paralysis, are commonly associated with neuronal dysfunction and are amenable for manipulation or scoring in a screen. As such, *C. elegans* is an excellent model organism for the modeling of ALS.

ALS is a complicated disease, with both genetic and environmental influences contributing to pathogenesis. Most cases are sporadic, but ∼10% are familial (Mejzini et al., 2019). To date, four genes have been identified that contribute to familial ALS: superoxide dismutase 1 (*SOD1*), transactive response DNA-binding protein (*TARDBP*), which produces the TDP-43 protein, fused in sarcoma (*FUS*), and an uncharacterized open reading frame on chromosome 9 named *C9orf72* (Boylan, 2015). Up to dozens of variants in each of these genes have been associated with ALS, making disease etiology and modeling challenging (Mejzini et al., 2019); however, certain variants are disproportionately represented in patients. Further, it remains unclear whether pathogenic variants result in loss- or gain-of-function toxic effects. Although *C. elegans* orthologs exist for each of these genes, the most productive work has been performed on transgenic animals expressing the human WT or variant forms of TDP-43 and FUS (Aggad et al., 2014; Ash et al., 2010; Murakami et al., 2012; Therrien and Parker, 2014; Vaccaro et al., 2012a,b, 2013).

**Fig. 4.**
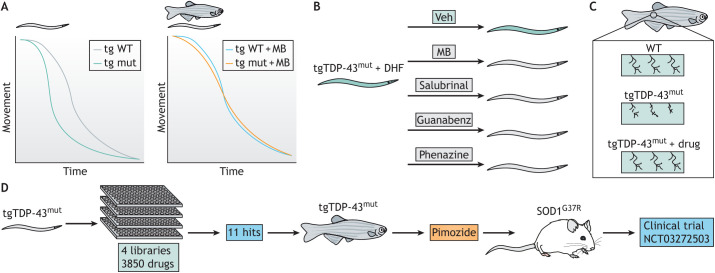
**Multi-system approach to the identification of pimozide as an amyotrophic lateral sclerosis (ALS) therapeutic.** (A) Stylized movement data of *C. elegans* and/or zebrafish with transgenic expression (tg) of WT TDP-43 or FUS or mutant (mut) TDP-43 or FUS. Transgenic expression of either mutant TDP-43 or FUS in *C. elegans* decreases movement (left). Treatment with Methylene Blue (MB) in either *C. elegans* or zebrafish rescues movement defects caused by transgenic expression of mutant TDP-43 or FUS (right) (Vaccaro et al., 2012a,b). (B) *C. elegans* expressing mutant TDP-43 (tgTDP-43^mut^) transgenes activate the fluorescent oxidative stress reporter dihydrofluorescein (DHF; green). Treating the transgenic animals with MB, salubrinal, guanabenz or phenazine reduced DHF fluorescence, indicating reduced oxidative stress (Vaccaro et al., 2012a, 2013). (C) Transgenic expression of mutant TDP-43 also induced neurodegenerative phenotypes in zebrafish, including axonal breaks, reduced branching and aberrant branching patterns indicated in the middle box. Similar to tgTDP-43^mut^ worms, these phenotypes were rescued by treatment with salubrinal, guanaben or phenazine (Vaccaro et al., 2012a, 2013). (D) The drug screening pipeline for discovery of pimozide, which advanced to a clinical trial (ClinicalTrials.gov NCT03272503). After the initial screen in *C. elegans* transgenically expressing mutant TDP-43, hits were validated in zebrafish, and pimozide was tested in the SOD1^G37R^ mouse model of ALS prior to advancing to clinical trial (Bose et al., 2019; Patten et al., 2017). Veh, vehicle.

Parker's group has either generated or used previously developed transgenic *C. elegans* strains expressing WT or dominant variant human TDP-43 and FUS under the control of both pan-neuronal and motor neuron-specific promoters (Ash et al., 2010; Murakami et al., 2012; Vaccaro et al., 2012b). Transgenic animals expressing the human variant, but not the human WT, forms of either protein faithfully recapitulated many of the ALS pathologies, including progressive motor dysfunction, neurodegeneration and ER stress (Ash et al., 2010; Murakami et al., 2012; Vaccaro et al., 2012b). Using whole-animal paralysis as their readout, Parker's group tested compounds with known neuroprotective effects and identified Methylene Blue (MB) for its ability to rescue the motility defects of the variant TDP-43 and FUS transgenic animals (Fig. 4A). Further analysis determined that MB had no effect on the WT transgenics at the effective concentration (Vaccaro et al., 2012a). This work was further validated in zebrafish with Parker's longtime collaborator Pierre Drapeau (Université de Montréal), thereby indicating the potential utility of this compound for ALS treatment in vertebrates (Vaccaro et al., 2012a). The identification of MB as a potential neuroprotective candidate for ALS is an important step forward in drug discovery for this disease.

Parker and colleagues used MB as a positive control in a subsequent drug discovery screen. They explored likely mechanisms of ALS pathogenesis, ER and oxidative stresses, and the negative effects of prolonged activation of the ER unfolded protein response (UPR^ER^). In this second screen, Parker's group successfully identified additional compounds such as salubrinal, guanabenz and phenazine as potent suppressors of the paralysis phenotypes in the TDP-43 variant transgenic animals (Fig. 4B) (Vaccaro et al., 2013). As with MB, these newly identified drugs were confirmed to also be effective in zebrafish ALS models (Fig. 4B) (Vaccaro et al., 2013). Mechanistic analyses revealed that these compounds alleviate the UPR^ER^, albeit through slightly different mechanisms compared to MB (Vaccaro et al., 2013). As such, these drugs could be used at lower doses in combination without loss of efficacy.

Building on their successes, Parker, Drapeau and Lawrence Korngut from the University of Calgary (Calgary, AB, Canada) screened nearly 4000 clinically approved compounds on transgenic *C. elegans* expressing variant TDP-43 (Patten et al., 2017). They identified a small group of neuroleptic compounds capable of rescuing the TDP-43-induced paralysis. Amongst these, pimozide had the most potent effect and, as with the previous candidate drugs, was validated in zebrafish (Fig. 4C). Pimozide provided neuroprotective effects for TDP-43 transgenic *C. elegans* when administered throughout the life of the animals. Any shorter treatment was not reported in *C. elegans*; however, overnight treatment of zebrafish with low-dose pimozide significantly improved multiple neurodegenerative phenotypes, including paralysis and neuronal breaks. Further analyses demonstrated that pimozide effectively provided neuroprotective benefits to SOD1 and FUS zebrafish models of ALS, indicating a genotype-independent effect (Patten et al., 2017), an important consideration for treatment of human disease.

Following the identification of pimozide in *C. elegans* and its acute effects in zebrafish, Parker and colleagues assessed the effects of pimozide in a SOD1 mouse model of ALS (Patten et al., 2017). This important preclinical study validated the neuroprotective efficacy of pimozide in a mammalian system and confirmed its benefits. The results from this study have resulted in a Phase 2 clinical trial for treatment of ALS patients with pimozide (Pimozide2; ClinicalTrials.gov NCT03272503; Fig. 4C). This clinical trial began in 2017 and completed at the end of 2020. No intermediate results have been reported at the time of writing this Review.

Despite the success leading to the identification of pimozide and its testing in a clinical trial, pimozide has side effects such as dizziness and gastrointestinal issues, making it a less than ideal treatment option for ALS patients. Thus, Parker, Drapeau and colleagues investigated pimozide derivatives. Using a similar approach to that employed for the identification of pimozide, nearly 3800 pimozide derivatives were screened in *C. elegans* with promising hits characterized in *C. elegans* and zebrafish, and confirmed in mice (Bose et al., 2019). This screen identified TRVA242 as a potent suppressor of motor neuron degeneration and dysfunction in each animal model (Bose et al., 2019). Although TRVA242 has not yet advanced to a clinical trial, it seems likely that it will be considered following the conclusion of the pimozide trial.

We highlight Parker's work not only because it displays a methodical and successful strategy for drug discovery, but also because it demonstrates a collaborative approach that used multiple model organisms to their greatest potential. Pimozide may not have been discovered for ALS treatment had it not been first identified in a *C. elegans* screen, and it certainly would not have reached a human clinical trial without the supporting evidence from zebrafish and mouse models. Together, these model organisms bring hope of a treatment to ALS patients.

### *C. elegans* for drug screens when there is no ortholog

The aforementioned amenability of *C. elegans* for high-throughput *in vivo* analyses also makes this nematode a powerful tool in the implementation of computational pharmacology. In some cases, researchers perform screens for drug targets upon expressing human genes that do not have corresponding nematode orthologs as transgenes in *C. elegans*. One example of this strategy is the *C. elegans* model for α1-antitrypsin deficiency (ATD). α1-antitrypsin (AT) is a secreted protease inhibitor encoded by the *SERPINA1* gene, the expression of which is enriched in the mammalian liver. The WT form of this protein is termed α1-antitrypsin M (ATM). However, a missense mutation in *SERPINA1* gives rise to the mutant α1-antitrypsin Z (ATZ), which misfolds and accumulates in cells. Cirrhosis and fibrosis of the liver are thought to develop as a result of the proteotoxic environment that follows ATZ accumulation in hepatocytes (Perlmutter, 2011).

*C. elegans* has no ortholog to human *SERPINA1*. The group led by Stephen Pak, Washington University in St. Louis (St Louis, MO, USA), expressed both the WT ATM and the disease-causing ATZ in the *C. elegans* intestine, the sole digestive cells in these nematodes. Analogous to what has been observed in humans, the ATM model displayed normal secretion of the protein into the intestinal lumen, whereas the ATZ disease model developed protein accumulation in the ER as well as increased autophagy in these cells (Gosai et al., 2010). Given its faithful recapitulation of disease phenotypes, this ATD model was used in a high-throughput, genome-wide RNAi screen to identify genes that, when depleted, significantly affected ATZ accumulation in the intestine of these animals (O'Reilly et al., 2014; Wang et al., 2019). This screening strategy identified 54 modifier genes, which were also verified orthologs of human genes. These genes were subsequently used for a clever drug discovery screen to identify potential ATD therapeutics.

**Fig. 5.**
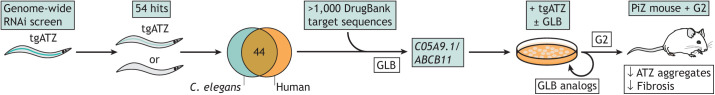
**Approach to identify the GLB analog G2 as a treatment for α1-antitrypsin deficiency (ATD).** Transgenic expression of green fluorescent protein (GFP)-tagged human α1-antitrypsin Z (ATZ), but not GFP-tagged human α1-antitrypsin M (ATM), in *C. elegans* results in GFP accumulation in intestinal cells, indicating protein aggregation (Gosai et al., 2010). A genome-wide RNA interference (RNAi) screen for suppressors of ATZ accumulation identified 54 hits that reduced the intensity of the GFP signal from accumulating GFP-tagged ATZ. Of these, 44 could be mapped to a human ortholog (Wang et al., 2019). Sequence comparison of the 44 genes with known drug targets from the DrugBankv3.0 library yielded *C05A9.1*/*ABCB11* as the best candidate, because it was solely targeted by the drug Glibenclamide (GLB). The ability of GLB to reduce ATZ accumulation was verified in mammalian cells transgenically expressing ATZ. Iterative testing of GLB analogs in these transgenic mammalian cells yielded G2 as a candidate for ATD treatment that avoided the insulin secretagogue effects of GLB. G2 was tested in the PiZ mouse model of ATD, resulting in reduced hepatic ATZ aggregates and fibrosis (Wang et al., 2019).

Pak's group mined DrugBankv3.0, a library of more than 1000 targets of FDA-approved drugs, to find ones predicted to affect the genes identified in the RNAi *C. elegans* screen. They compared the list of human orthologs to the *C. elegans* modifiers with the drug targets and, when extrapolated back to *C. elegans*, identified three *C. elegans* genes with high sequence similarity to known drug targets. Analysis of the function of these genes and their human orthologs led Pak's group to focus on the *C. elegans* gene *C05A9.1* and its human ortholog *ABCB11*. The ABCB11 protein was of particular interest because, according to DrugBankv3.0, it was targeted by a single drug, Glibenclamide (GLB) (Fig. 5) (Wang et al., 2019). GLB is a sulfonylurea compound and has previously been approved for treatment of type 2 diabetes mellitus as it promotes secretion of insulin from pancreatic β cells (Proks et al., 2002). GLB has FDA approval, which should improve its availability for off-label use to treat ATD. However, the potential for complications from increased insulin secretion posed a challenge. Pak and colleagues needed to separate any ATD-associated activity from its insulin secretagogue effects.

Similar to Parker's work described above, Pak's group validated their findings in other model systems. Analyses in multiple human cell lines allowed the researchers to confirm that GLB treatment increased degradation of ATZ aggregates by the autophagolysosomal system (Gosai et al., 2010; Wang et al., 2019). To separate the autophagolysosomal effects from GLB's known insulin secretagogue effects, three analogs of GLB were further assessed for their capacity to improve ATZ degradation without stimulating insulin secretion. Assays in multiple human and murine cell lines, including the Min6 mouse pancreatic β cell line, demonstrated that two of the three GLB analogs effectively promoted ATZ degradation without inducing insulin secretion (Wang et al., 2019). Finally, Pak's group confirmed the efficacy of the GLB analog G2 in the PiZ mouse model of ATD. G2 treatment improved clearance of hepatic ATZ aggregates and reduced fibrosis without affecting glycemia (Fig. 5B) (Wang et al., 2019). These observations are exciting, and we look forward to the further studies and potential clinical trials of this GLB analog.

Early studies in *C. elegans* provided the foundation for the identification, investigation and validation of G2 for ATD treatment despite the lack of an ortholog for the disease-causing gene in *C. elegans*. Similar strategies can be adopted for other diseases and will continue to foster collaboration between nematode biologists and the field of pharmacology.

## Conclusions

The studies discussed in this Review represent just a few of the many examples of how *C. elegans* is being utilized to study the mechanisms of rare diseases. Additional examples are summarized in Table 1. From basic understanding of protein function to drug screens resulting in clinical trials, *C. elegans* have demonstrated their value in understanding human health. Although the studies presented here rely heavily on transgenic expression of variant forms of genes/proteins, we foresee CRISPR/Cas9 editing becoming the favored tool of genetic manipulation in the future. For many rare diseases, *C. elegans* has an ortholog that can be edited to contain the analogous mutation observed in the individuals affected. Moreover, the ATD deficiency example highlights the possibility of studying dominant mutations in human genes that do not have a *C. elegans* ortholog. Such studies also reveal the utility of high-throughput drug screening to identify compounds that can suppress phenotypes caused by the expression of a given variant. Overall, it is the collaborative nature of these studies that gives greater meaning to the scientific endeavor. We present here multiple examples of collaborations across model systems, as well as work that bridges the gap between the bench and the clinic. Without the clinicians identifying the rare diseases and the many different model system labs focusing on them, progress for therapies and treatments would be significantly hampered. As both diagnostic and investigative technologies continue to improve, we are sure that disease modeling in *C. elegans* will continue to contribute to our understanding and treatment of human rare diseases.

**Table 1.**
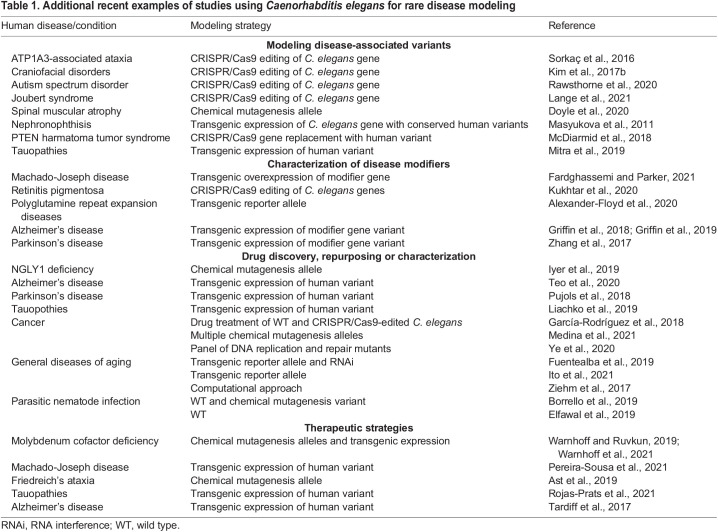
Additional recent examples of studies using *Caenorhabditis elegans* for rare disease modeling
